# Classification epitopes in groups based on their protein family

**DOI:** 10.1186/1471-2105-16-S19-S7

**Published:** 2015-12-16

**Authors:** Edgar Ernesto Gonzalez Kozlova, Benjamin Thomas Viart, Ricardo Andrez Machado de Avila, Liza Figueredo Felicori, Carlos Chavez-Olortegui

**Affiliations:** 1 Laboratório de Imunoquímica de Proteínas, Departamento de Bioquímica-Imunologia, Instituto de Ciências Biológicas, Universidade Federal de Minas Gerais, CP: 486; CEP: 31270-901, Belo Horizonte-MG, Brazil; 2 Laboratório de Biologia Celular e Molecular, Programa de Pós-Graduação em Ciências da Saúde, Unidade Acadêmica de Ciências da Saúde, Universidade do Extremo Sul Catarinense, CEP: 88806-000. Criciúma-SC, Brazil

**Keywords:** >Data mining, B cell epitopes, metalloproteinases, neurotoxins, protein family, epitope prediction

## Abstract

**Background:**

The humoral immune system response is based on the interaction between antibodies and antigens for the clearance of pathogens and foreign molecules. The interaction between these proteins occurs at specific positions known as antigenic determinants or B-cell epitopes. The experimental identification of epitopes is costly and time consuming. Therefore the use of *in silico *methods, to help discover new epitopes, is an appealing alternative due the importance of biomedical applications such as vaccine design, disease diagnostic, anti-venoms and immune-therapeutics. However, the performance of predictions is not optimal been around 70% of accuracy. Further research could increase our understanding of the biochemical and structural properties that characterize a B-cell epitope.

**Results:**

We investigated the possibility of linear epitopes from the same protein family to share common properties. This hypothesis led us to analyze physico-chemical (PCP) and predicted secondary structure (PSS) features of a curated dataset of epitope sequences available in the literature belonging to two different groups of antigens (metalloproteinases and neurotoxins). We discovered statistically significant parameters with data mining techniques which allow us to distinguish neurotoxin from metalloproteinase and these two from random sequences. After a five cross fold validation we found that PCP based models obtained area under the curve values (AUC) and accuracy above 0.9 for regression, decision tree and support vector machine.

**Conclusions:**

We demonstrated that antigen's family can be inferred from properties within a single group of linear epitopes (metalloproteinases or neurotoxins). Also we discovered the characteristics that represent these two epitope groups including their similarities and differences with random peptides and their respective amino acid sequence. These findings open new perspectives to improve epitope prediction by considering the specific antigen's protein family. We expect that these findings will help to improve current computational mapping methods based on physico-chemical due it's potential application during epitope discovery.

## Background

Living organisms often encounter a pathogenic virus, microbe or any foreign molecule during it's lifetime [1]. The B cells of the immune system recognize the foreign body or pathogen's antigen by their membrane bound immunoglobulin receptors, which later produce antibodies against this antigen [2,3]. The recognized sites on the antigen's surface, known as epitopes, represent the minimum wedge recognized by the immune system [4]. Therefore, epitopes lie at the heart of the humoral immune response [5]. The rapid reaction to a previously encountered antigen depends on the binding ability of the antibodies found in the immune system of the organism [6], the physico-chemical properties of the epitope and it's structural conformation [7]. Thus, understanding epitope characteristics and how they are recognized, in sufficient detail, would allow us to identify and predict their position in the antigen [8].

The main objective of epitope prediction is to design a molecule that can replace an antigen in the process of either antibody production or antibody detection [4,9-11]. Such a protein can be synthesized in case of peptides or in case of a larger protein, produced by yeast after the gene is cloned into an expression vector [12]. After 30 years of research, it is known that the optimum size of peptides possessing cross-reactive immunogenicity is between 10-15 amino acids [13]. The earliest efforts made to understand and predict B-cell epitopes were based on the amino acid properties, such as flexibility [14], hydrophaty [15], antigenicity [7], beta turns [16] and accessibility [17]. Epitope prediction is important to design epitope-based vaccines and precise diagnostic tools such as diagnostic immunoassay for detection, isolation and characterization of associated molecules for various disease states. These benefits are of undoubted medical importance [18,19].

Recently developed prediction methods face several challenges like data quality [20,7], a limited amount of positive learning examples [21] or difficulty in choosing an appropriate negative learning examples [22]. These negative training samples may harbor genuine B cell epitopes and affect the training procedure, resulting in a poor classification performance [23,24]. Moreover, none of the published work took into account the protein family or function to predict epitopes [25].

The present study explores the possibility of epitopes belonging to same protein family share common properties. For these purpose, the amino acid statistics, physico-chemical and structural properties were compared within each other [26] for two protein's group. This assumption is based on previous studies showing that it exists amino acid trends in composition and shared properties for intravenous immunoglobulins [27]. Despite the difficulty of distinguishing epitopes from non epitopes [28] the addition of information, such as evolutionary and propensity scales, proved to be helpful for epitope prediction [21]. Therefore, it is interesting to assume including information about the protein antigen's family may be resourceful to improve prediction.

## Methods

### Dataset composition

We have obtained experimentally validated 106 linear B-cell epitopes for two groups of antigens (metalloproteinases and neurotoxins) extracted from Pubmed (http://www.ncbi.nlm.nih.gov/pubmed/).

They were manually curated until September 2012 following several search criteria based on the keywords: epitope, metalloproteinase, proteinase, peptidase, toxin and neurotoxin in a joint and disjoint manner. The redundancy was removed for repeated sequences using 100% identity as threshold and the maximum size of the epitopes was fixed to be equal or less than 32. As non epitope data, we created 49 linear random peptides proportional number to the mean of the amount of epitopes in the groups metallorproteinase and neurotoxin. These random peptides are based on the statistics from the dataset UniProtKB/Swiss-Prot, meaning that the sum of the random peptides amino acids are equal to the percentages found in uniprot database. The final set contained 99 non redundant epitopes, containing 29 metalloproteinases, 70 neurotoxins and 49 random peptides as showed in Additional file 1.

### Feature selection for data mining analysis

In this study, we generated and used 33 physico-chemical parameters composed by aliphatic index, GRAVY, isoelectric point, amino acid content in percentages, amino acid groups such as hydrophobic (AVILMFYW), positive charged (RHK), negative charged (DE), not charged (STNQ) and specials (SGP) as described by Gasteiger with the difference that each feature was transformed to percentage removing the length difference for the epitope sequences [29]. Also 6 predicted secondary structure properties such as strand, helix, coil, relative surface accessibility, absolute surface accessibility and z-fit which were calculated with Netsurf algorithm [29]. These parameters were calculated for the three groups in study (Metalloproteinase, Neurotoxin and Random) and the results where compared using Welch two sample t-test available in the statistical software R. In total, we evaluated 3 different matrices for the classification purpose of discover how much sequence-derived information was needed to obtained a good classification. The first matrix based of purely PCP information, a second with only PSS data and a third one which was merely the addition of the PSS features to the PCP matrix.

### Selection of data mining methods and statistical analysis

The Konstanz Information Miner (KNIME) [30] was used to evaluate Kmeans (KM), decision tree [31] (DT), naive bayes classifier (NB), support vector machine [32] (SVM) for the matrices generated with our dataset. The free software environment R for statistical computing and graphics was used to create the multiple regression models (LMR). For LMR the nominal class variable was transformed into a numerical variable for the two groups, a positive with value log(0.99/(1-0.99)) for metalloproteinases and a negative been log(0.01/(1-0.01)) for neurotoxins. The linear model function available in R was used to solve a series of equations where the class variable was equal to the feature variables. After solving the equations, a linear multiple regression model was generated, a p-value was calculated and the model was rejected for any p-value superior to 0.005. The predicted resulting score of the model was scaled (0 to 1) by using exp(predicted value./(1+predicted value)) formula. The performance of all the generated models was evaluated for every possible decision threshold with ROCR package by using the parameters AUC (area under the curve formed by true and false positive rates) and accuracy, which gives an overall view of the performance of the classification method used [33].

## Results

### Statistical differences of amino acid composition between metalloproteinase and neurotoxin linear epitopes compared with random sequences

The dataset contain 11 metalloproteinases and 16 neurotoxins. The two protein families (or group) respectively contains 29 and 70 epitopes with an average sequence length of 13.8 amino acids (aa). The minimum length was 4 aa and maximum 32 aa. The negative or non epitope set contained 49 sequences of 14 aa length (Table 1).

**Table 1:** Dataset composition

Groups	Proteins	Epitopes	Non epitopes
Uniprot	544996	--	--
Neurotoxin	16	29	0
Metalloproteinase	11	70	0
Negative examples	13	0	49

The metalloproteinase and neurotoxin epitopes showed to be different from each other showing a statistical dissemblance for a confidence interval of 95% for the amino acids R, K, M and Y (Table 2, column 1). Also when compared these epitopes to their respective proteins they showed differences for the amino acids R, Q, V and M for metalloproteinases (Table 2, column 4) and D and C for neurotoxins (Table 2, column 5).

These epitope groups also indicated variation when compared to our non epitope control for the amino acids K, C, A, V and I for metalloproteinases and R, K, D, N, Q, C, A, I, K, M and W for neurotoxins (Table 2 columns 2 and 3). As expected, we also detected differences in other parameters such as aliphatic index, grand average of hydropaty and isoelectric point (Table 2 last three rows). Therefore, we were able to identify common characteristics in epitope's composition within unique antigen groups and differences between neurotoxin and metalloproteinase epitope groups.

**Table 2:** Analysis of means for all datasets with Welch two sample T-test

Parameter	p - values for a confidence interval of 95%				
	
	(1)ME vs NE	(2)Random vs ME	(3)Random vs NE	(4) MP vs ME	(5) NP vs NE
R (Arg)	**0.0029**	0.0762	**0.0001**	**0.0241**	0.4226
H (His)	**0.0362**	0.1046	0.1074	0.5636	0.7906
K (Lys)	**0.0000**	**0.0113**	**0.0000**	0.4098	0.4818
D (Asp)	0.0890	0.6994	**0.0079**	0.7091	**0.0030**
E (Glu)	0.9289	0.2681	0.0838	0.6696	0.4072
S (Ser)	0.2953	0.5024	0.3546	0.9630	0.8954
T (Thr)	0.4077	0.1867	0.3509	0.2199	0.4523
N (Ans)	0.1878	0.7647	**0.0101**	0.5880	0.4944
Q (Gln)	0.1509	0.9483	**0.0039**	0.8471	0.8185
C (Cys)	0.1821	**0.0003**	**0.0000**	**0.0316**	**0.0075**
G (Gly)	0.6979	0.2576	0.4620	0.3509	0.8450
P (Pro)	0.3156	0.5165	0.3781	0.2103	0.4271
A (Ala)	0.2121	**0.0066**	**0.0000**	0.1092	0.0756
V (Val)	0.0993	**0.0019**	0.2903	0.0550	0.1854
I (Ile)	0.2657	**0.0068**	0.0352	0.1286	0.3275
L (Leu)	0.1374	0.1182	**0.0000**	0.5549	0.2322
M (Met)	**0.0017**	0.0725	**0.0000**	**0.0282**	0.2477
F (Phe)	0.6997	0.4713	0.0765	0.7890	0.5818
Y (Tyr)	**0.0023**	0.5245	**0.0000**	0.8318	0.0938
W (Trp)	0.0889	0.9443	**0.0244**	0.5782	0.1221
Isoe.Point	0.0425	0.5190	0.5190	**0.0425**	0.3221
gravy	0.0672	**0.0010**	**0.0000**	0.0672	**0.0514**
Aliph. Index	**0.0086**	**0.0000**	**0.0000**	**0.0086**	0.8550

Values under p-value under 0.05 are writen in bold. IC = 95%, H0 = Difference in means is cero. Hi = Difference in means is not equal to zero. Metalloproteinases epitopes = ME, Neurotoxin epitopes = NE, Metalloproteinase proteins = MP, Neurotoxin proteins = NP, Random = Random sequences.

### Decision tree and multiple regression models can distinguish linear B-cell epitopes from two different antigen groups

We investigated our capacity to discriminate if an epitope belonged to neurotoxin or metalloprotease based on the statistical significant differences observed in epitopes amino acids composition, isoeletric point, gravy and aliphatic index (Table 2). For this purpose, we used five different methods: SVM, NB, DT, KM and LMR.

Our analysis used three different input matrices as described before: Only physico-chemical properties (PCP), only secondary structure (PSS) and the combination of both (PCP+PSS) for each algorithm. The performances displayed as AUC values for all data mining methods are showed in table 3. All the methods with the exception of KM were able to group and distinguish correctly both groups of epitopes. As expected, the best results were for SVM followed by similar performance by much simpler techniques, LMR and DT.

**Table 3:** Performance of all data mining methods showed in AUC and accuracy.

Matrix	PCP		PSS		PCP+PSS	
**Models**	**AUC**	**Accuracy**	**AUC**	**Accuracy**	**AUC**	**Accuracy**

SVM	1	1	1	1	1	1
MLR	0.986	0.952	0.655	0.714	1	1
DT	0.957	0.962	0.921	0.943	0.943	0.952
NB	0.8	0.838	0.521	0.667	0.793	0.838
KM	0.493	0.667	0.509	0.681	0.507	0.667

During the use of PSS features as input, a reduction in the performance of 0.1-0.3 AUC value was noticed for MLR and NB techniques (Table 3). Only SVM and DT obtained an AUC superior to 0.9 while all the other methods performed poorly with AUC of 0.65 for LMR and close to 0.5 for the others. The SVM technique performed with an AUC of 1.0 for combined properties while LMR showed a slight increase from 0.9 to 1.0. By the other hand DT, NB and Kmeans stayed the same (Table 3). These results indicate that the type of input used (PSS or PCP) were not significant, where the models based on the PCP were the simplest to analyze and understand. The most stable AUC results were obtained with DT method where all the matrices analyzed resulted in an AUC value around 0.95.

The techniques DT and LMR are statistical approaches that showed results similar to SVM which is a non statistical classifier. These methods allowed us to discriminate the epitopes belonging to metalloproteinases or neurotoxins and to identify the important properties inside these groups. The relevant features to classify the epitope groups for the LMR and DT models can be found in table 4.

**Table 4:** Properties used by the classification models until 8º order out of 39.

	Classification Model: Linear Multiple Regression		
**Order**	**PCP**	**PSS**	**PCP+PSS**
1º	Statistic of N	Z-fit	Statistic of E
2º	Statistic of Q	ASA	Statistic C Atoms
3º	Statistic of S	RSA	Statistic of N
4º	Statistic of T	Strand index	Statistic of Q
5º	Uncharged STNQ	Helix index	Statistic of S
6º	Special CGP	Coil index	Statistic of T
7º	Statistic H Atoms	--	Uncharged STNQ
8º	Statistic C Atoms	--	Statistic H Atoms

	**Classification Model: Decision Tree**		

**Order**	**PCP**	**PSS**	**PCP+PSS**
1º	Statistic of K	Z-fit	Statistic of K
2º	Statistic of D	RSA	Statistic of D
3º	Statistic of M	ASA	Statistic of M
4º	Statistic S Atoms	Strand index	Statistic S Atoms
5º	Statistic of I	Coil index	Statistic of I
6º	Statistic of W	--	Statistic of W
7º	Statistic of Y	--	Coil index
8º	Isoelectric point	--	--

We observed which amino acids were critical to differentiate epitopes from neurotoxins and metalloproteinases. In the case of LMR model, the amino acids asparagine (N), glutamine (Q) and serine (S), and in the case of DT model the amino acids lysine (K), aspartate (D) and methionine (M) were the key to achieve good classification (above 0.9 AUC) (Table 4).

## Discussion

The amino acid composition has been investigated for proteins related to the B-cell response [34] and as key for understanding protein-protein interactions [35,36] alongside their role during prediction of epitopes for both T and B-cells [37]. Epitopes are rich in charged and polar amino acids and low in aliphatic hydrophobic amino acids, when comparing the epitope amino acid distribution to either the entire PDB database [38] or to the antigen [39,40]. Also Rubinstein [39] suggested that the amino acid Tyr is significantly over-represented in epitopes and that Val is significantly depleted. Interestingly, the residues Arg and Lys are more frequent in the epitopes of our dataset along other differences as aliphatic index and gravy. This particularities are probably a result of focusing common features in a diverse epitope group, phenomena which was evidenced in the amino acids composition found in epitopes for papilloma viruses [22]. The PCP based methods have been explored in detail for epitope prediction [40] with some limitations in terms of specificity and precision as seen in models for SVM with AUC values of 0.85 for amino acid composition and 0.58, where the accuracy never surpass 0.8 [26].

Our study suggests an improvement in performance when a single epitope group is targeted, resulting in AUC and accuracy superior to 0.9. We included groups of amino acids based on type of charge and lateral chain due to the the concept of amino acids working cooperatively in protein:protein interfaces [41]. Our results indicate that these amino acid groups such as hydrophobic, polar, or special amino acids (CGP), do not posses significance for the prediction models by themselves but may add value when combined with single amino acid statistics.

The secondary structure of epitopes was also investigated by several authors [42-44], and epitopes are in general reported to have significantly less strands and helices and significantly more loops compared to the rest of the antigen [8,38]. The over-representation of loops is small but significant and in agreement with the perception that protein-protein binding sites are flexible regions [41]. The overall secondary structure of epitopes has been reported to been different from regular protein-protein interfaces [23] based on crystals available on the PDB indicating some structural particularities of the Ab-Ag interaction [45]. These particularities could be also family restrictred which could be interesting to explore with computational methods despite of having an accuracy of 79% when predicted from sequence [46] but the DT outcome showed no real relevance in PSS features when applied to epitope classification. The inclusion of predicted secondary structure as commonly done [40] could be a source of misleading results for the prediction, issue which has been reviewed briefly in the literature [47].

The features that characterize each epitope's group could represent the complementary data needed to improve epitope prediction. For example, when adding evolutionary information to the prediction the performance was improved [48] despite recent studies that explain no relation exits between epitope and antigens sequences [28]. Therefore, we showed that a wide range of data mining methods including support vector machine [21], decision tree [48], regression [26] and Naive Bayer classifier had similar successful results bringing some light to the question of which characteristics are important for these epitope groups. It's important to note that we used amino acid percentage [4] in comparison with some recent epitope prediction methods that prefer propensities [12]. The data normalization made in the present study are based on the assumption that each feature is equally relevant for any protein sequence based analysis [9]. We also demonstrate that despite the method, it was possible to classify the studied groups, pointing out the importance of the quality of the used data [49].

## Conclusions

Our study indicates that linear epitopes that belong a single protein family share common properties but different when compared to epitopes from different families, as demonstrated for neurotoxins and metalloproteinases. We confirmed our hypothesis with five different data mining algorithms, probabilistic and non probabilistic, showing similar results except for Kmeans. The proposed models allowed to separate the studied groups from random sequences based on Uniprot statistics. The models based only in PCP features were enough to show and identify the differences between epitope groups. Therefore, we demonstrate that considering the epitope's protein family can reveal unseen patterns within epitope groups that could be used to improve epitope discovery.

## List of abbreviations

SVM: Support Vector Machine

NB: Naive Bayes

DT: Decision Tree

KM: K-Means

LMR: Linear Multiple Regression

PDB: Protein Data Bank

PSS: Position Specific Matrix

PCP: Physico-Chemical-Properties

ASA: Absolute Surface Area

RSA: Relative Surface Area

AUC: Area Under the Curve

ROC: Receiver Operating Characteristic

ME: Metalloproteinase epitopes

MP: Metalloproteinase proteins

NE: Neurotoxin epitopes

NP: Neurotoxin proteins

## Competing interests

The authors declare that they have no competing interests.

## Authors' contributions

Carlos Chavez Olortegui: Advising, professional orientation, results review and science encouragement.

Edgar Ernesto Gonzalez Kozlova: Data mining models and statistical analysis.

Benjamin Thomas Viart: Statistical analysis advising.

Liza Figueredo Felicori: Hypothesis help and advising.

Ricardo Andrez Machado de Avila: Hypothesis help and advising, general advising, results review and science encouragement.

## Supplementary Material

Aditional file 1 **The datasets composed of the sequences used in this work is available in this .csv file, containing four columns**. First column shows the pubmedID of the paper from which the sequence was extracted. The second column contains the sequence. The third collumn contain the sequence IDs from genebank, uniprot or pdb, databases. The fourth column contains the class of the sequences which can be neurotoxin, metalloproteinase or random. The column separator in this .csv file is a standart semicolon ";". Click here for file

## Declarations

This research and funding for publication was supported by Coordenação de Aperfeiçoamento de Pessoal de Nível Superior, (CAPES-Brazil),(Toxinologia No 23038000825/2011-63). Fundação de Amparo a Pesquisa do Estado de Minas Gerais, Brazil (FAPEMIG-Brazil) and *Conselho Nacional *de *Desenvolvimento *Científico e Tecnológico (CNPq-Brazil).

This article has been published as part of *BMC Bioinformatics *Volume 16 Supplement 19, 2015: Brazilian Symposium on Bioinformatics 2014. The full contents of the supplement are available online at http://www.biomedcentral.com/bmcbioinformatics/supplements/16/S19
